# Distinct effectiveness in containing COVID-19 epidemic: Comparative analysis of two cities in China by mathematical modeling

**DOI:** 10.1371/journal.pgph.0000043

**Published:** 2021-11-12

**Authors:** Yunpeng Ji, Pengfei Li, Qinyue Zheng, Zhongren Ma, Qiuwei Pan

**Affiliations:** 1 Key Laboratory of Biotechnology and Bioengineering of State Ethnic Affairs Commission, Biomedical Research Center, Northwest Minzu University, Lanzhou, China; 2 Department of Gastroenterology and Hepatology, Erasmus MC-University Medical Center, Rotterdam, The Netherlands; 3 School of Management, Shandong Key Laboratory of Social Supernetwork Computation and Decision Simulation, Shandong University, Jinan, China; Boston University, UNITED STATES

## Abstract

For better preparing future epidemic/pandemic, important lessons can be learned from how different parts of China responded to the early COVID-19 epidemic. In this study, we comparatively analyzed the effectiveness and investigated the mechanistic insight of two highly representative cities of China in containing this epidemic by mathematical modeling. Epidemiological data of Wuhan and Wenzhou was collected from local health commission, media reports and scientific literature. We used a deterministic, compartmental SEIR model to simulate the epidemic. Specific control measures were integrated into the model, and the model was calibrated to the recorded number of hospitalized cases. In the epicenter Wuhan, the estimated number of unisolated or unidentified cases approached 5000 before the date of city closure. By implementing quarantine, a 40% reduction of within-population contact was achieved initially, and continuously increased up to 70%. The expansion of emergency units has finally reduced the mean duration from disease onset to hospital admission from 10 to 3.2 days. In contrast, Wenzhou is characterized as an emerging region with large number of primarily imported cases. Quick response effectively reduced the duration from onset to hospital admission from 20 to 6 days. This resulted in reduction of *R* values from initial 2.3 to 1.6, then to 1.1. A 40% reduction of contact through within-population quarantine further decreased *R* values until below 1 (0.5; 95% CI: 0.4–0.65). Quarantine contributes to 37% and reduction of duration from onset to hospital admission accounts for 63% to the effectiveness in Wenzhou. In Wuhan, these two strategies contribute to 54% and 46%, respectively. Thus, control measures combining reduction of duration from disease onset to hospital admission and within-population quarantine are effective for both epicenters and settings primarily with imported cases.

## Introduction

The global population is constantly facing threats of emerging infectious diseases. Different countries and regions often react differently in response to outbreaks, whereas the right early response is essential for containing the outbreak, thereby avoiding large epidemic or pandemic. Because of their disparities in culture, socioeconomic status, and types of government, the implementation of control measures can vary tremendously among different countries.

For better preparing future epidemic/pandemic, important experiences and lessons could be learned from how the COVID-19 epidemic was responded from different regions of China, in particular at their different stages. COVID-19 was sparked in December, 2019 from Wuhan, the capital of Hubei province, with a population of 11 million [1]. Due to initial delay of necessary actions, the local government missed the first chance of containing the epidemic. Until January 23, the central government imposed heavy control measures, including city lockdown, travel ban, and within-population quarantine. The total confirmed cases has finally climbed up to nearly 50,000 cases in Wuhan, but was controlled subsequently.

COVID-19 outbreak was coincided with a massive population migration, because of the Chinese lunar new year holiday [2]. This has led to rapid spread across China. Of particular relevance is Wenzhou, a prefecture-level city of Zhejiang province with a total population of 9 million. It is 900 km away from Wuhan, and about 170,000 Wenzhou businesspeople are working there [3]. The first case in Wenzhou was identified on January 21, who returned from Wuhan. It quickly became the highest figure of COVID-19 cases for any city outside of Hubei province, and most of these cases were imported from the epicenter [3]. However, it took only about 46 days for this city to fully contain the epidemic, otherwise bearing high risk of growing into a new epicenter.

The distinct experiences of these two cities are mirroring what has happened in many parts of the world, either as epicenters or regions primarily with imported case. In this study, we aim to comprehensively compare the effectiveness of containing COVID-19 between Wuhan and Wenzhou by mathematical modeling, and to provide mechanistic understanding of how to effectively contain the epidemic at early stage.

## Methods

### Data collection

We systematically collected the epidemical data on SARS-CoV-2 transmission in Wuhan and Wenzhou. For Wuhan (S1 Table in S1 File), data regarding onset distribution of early identified cases before January 15, and number of cumulative and hospitalized cases were collected from the Health Commission of Hubei Province and previous studies [4–6]. For Wenzhou (Table 1), we collected data of all identified cases recorded by the Health Commissions of both Zhejiang Province and Wenzhou city [3].

**Table 1:** Parameters used in the quarantine simulation of Wenzhou epidemic.

Parameter	Symbol	Time	Baseline value	Estimated value	Reference
Contact rate	*c*	3–20 Jan	10	-	Assumption
		21–26 Jan	10	-	Assumption
		27–31 Jan	10	-	Assumption
		1–17 Feb	-	6	Estimated
Mean duration from onset to hospital admission	*i*	3–20 Jan	20 days		the Health Commission of Wenzhou
			(18–21)		
		21–26 Jan	12 days	-	*Li, et al. 2020, NEJM
			(10–14)		
		27–31 Jan	6.1 days	-	the Health Commission of Wenzhou
			(5–8)		
		1–17 Feb	6.2 days	-	the Health Commission of Wenzhou
			(5–8)		
Mean Recovery rate	*v*	-	0.045	-	the Health Commission of Wenzhou
			(0.04–0.066)		
Mean interval of incubation	*p*	-	5.5 days	-	the Health Commission of Wenzhou
			(3–7)		
Case fatality rate	*m*	-	0	-	the Health Commission of Wenzhou
Duration of quarantine	*r*	-	14 days	-	WHO recommendation

*Note: Li, Q., et al. (2020). Early Transmission Dynamics in Wuhan, China, of Novel Coronavirus-Infected Pneumonia. N Engl J Med. Mar 26;382(13):1199–1207.

### Model structure and control measures

**Fig 1:**
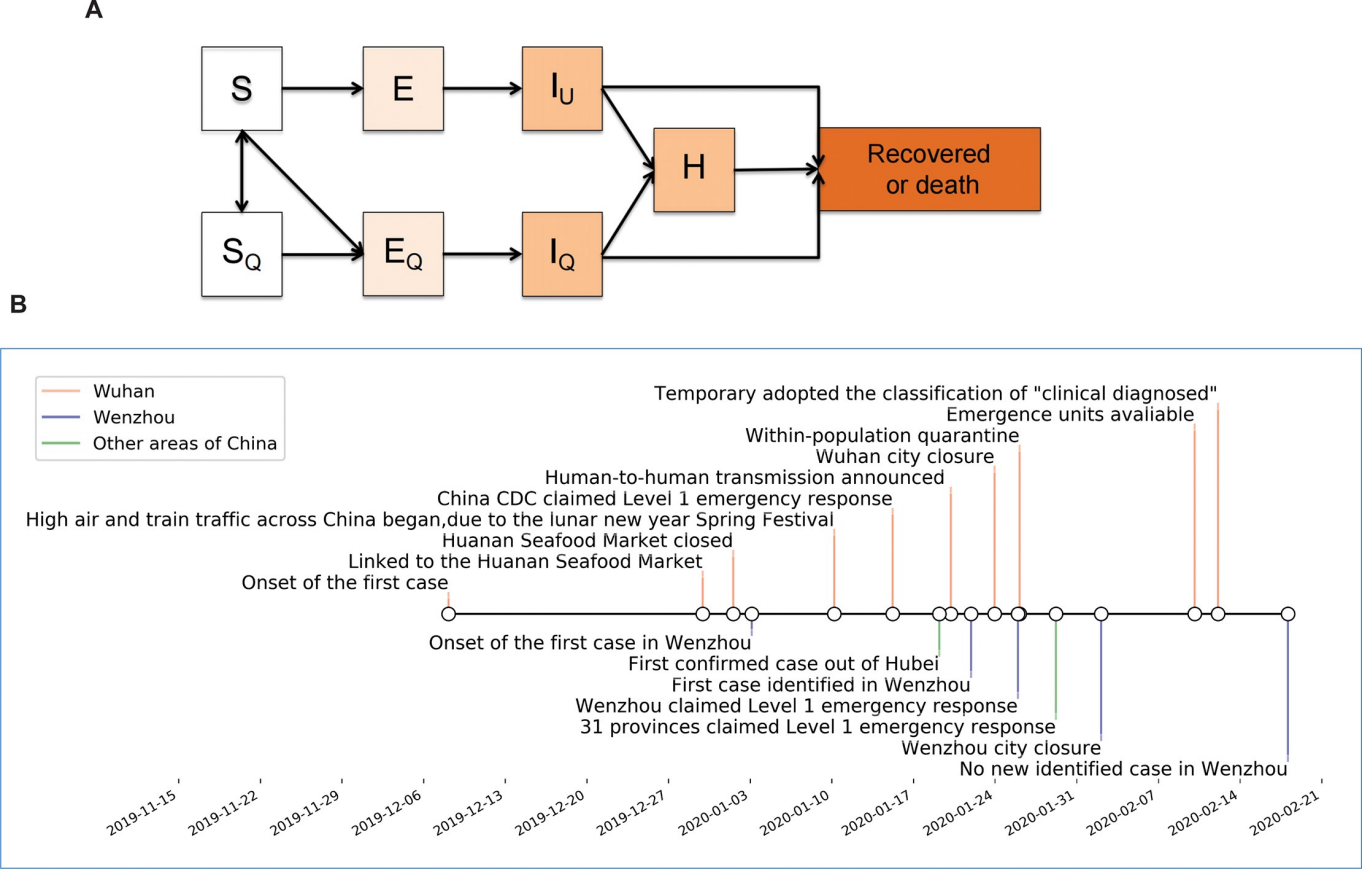
(A) Mathematical model for COVID-19 transmission. Susceptible individuals are infected by unidentified individuals, and become infectious themselves after an interval of incubation. Infectious individuals lose infectiousness by death, recovery, or successful isolation. No new births or deaths of other causes are considered. When quarantine started, a proportion, *q*, of new infections is quarantined before they become infectious. The same proportion (*q*) of susceptible individuals who have contacts with infectious persons but were not infected are also quarantined. Susceptible individuals are released from quarantine after 14 days. For simplicity, we assume that quarantined individuals are isolated and not infectious. (B) Timeline of important events occurred during COVID-19 outbreaks in Wuhan and Wenzhou.

We focused on two major control measures, isolation of symptomatic cases and reduction of within-population contact rates. To estimate the impact of the measures, we constructed a deterministic, compartmental model for SARS-CoV-2 transmission, in which a standard susceptible-exposed-infectious-recovered (SEIR) structure was modified to accommodate quarantine and isolation (Fig 1A). The model was fitted to data on hospitalized cases (*H(t))*) in Wuhan and Wenzhou from early January to February 2020. The model incorporates data on city population size, the current knowledge of natural history of SARS-CoV-2, and other relevant parameters.

Briefly, we assumed that all the citizens are initially susceptible (*S*), and a fraction *q* of all persons infected by an infectious case is successfully quarantined (*E*_*Q*_), and a fraction *q* of all persons contacted but not infected by an infectious case is also quarantined (*S*_*Q*_). The infectious compartment is composed of cases who are unidentified or unisolated (*I*_*U*_), those who develop from *E*_*Q*_ (*I*_*Q*_), and those who have been hospitalized (*H*). We assumed that cases in *I*_*Q*_ and *H* are successfully quarantined and would not infect other susceptible people.

We assumed that an infectious individual has a mean of *c* potentially infectious contacts per day, that susceptible contacts are infected with probability *β*, and that the number of contacts was independent of population density. We further assumed that individuals are hospitalized and correspondingly isolated at a fixed rate per day after becoming infectious, and that isolated individuals are no longer at risk of transmitting the virus. Infected individuals become noninfectious by dying, recovering, or being hospitalized, and the mean duration of infectiousness is *D* days.

We used least-square fitting to look for the model trajectory that best matches the epidemic time series and to estimate the parameters. Specifically, we fit the hospitalized number of cases given by equation *H(t)* to the hospitalize number of case notifications. Sets of realizations of the best-fit curve *H(t)* were generated using parametric bootstrap [7], in which 200 realizations were made. Each realization of the cumulative number of case notifications *H_i_*(*t*)(*i* = 1, 2,…,*m*) is generated as follows: for each observation *C*(*t*) for *t* = 2, 3,…,*n* days generates a new observation $H_{i}^{*}(t)\;for\;t\geq 2\;((H_{i}^{*}(1)=C(1))$ that is sampled from a Poisson distribution with mean *H*(*t*)−(*t*−1) (the daily increment in *H(t)* from day *t* -1 to day *t*). The corresponding realization of the cumulative number of infection notifications is given by $H_{i}(t)=\sum_{j=1}^{t}H_{i}^{*}(t)$, where *t* = 1, 2, 3,., n. Detailed methods for estimating *R*_*0*_ and *R*_*t*_ were described in S1 File.

### Estimating control measures in Wuhan and Wenzhou

Control measures were introduced with different time points (Fig 1B). In the model of Wuhan epidemic, we started on January 15, on which China CDC claimed to execute level 1 emergence. We set two time points. One is around January 27 when within-population quarantine was implemented, which would reduce the within-population contact rate. Another is around February 10, since then the newly built emergency medical units were operational. It mainly accelerates isolation of the unidentified cases. To evaluate the measures, we arbitrarily delayed the time point by 7 days and analyzed the effect. Seeds of the model were from the early 370 identified cases in Wuhan, because those were firstly proved to be associated with human-to-human transmission [4].

For Wenzhou, the sources of SARS-CoV-2 transmission were all imported cases with travel history to Wuhan or other areas of China. We started the model on the date of onset of the first imported case (January 3), and took all imported cases as model seeds. The first time point was set on January 21 as identification and hospitalization of cases were started. The second and third time points were set based on the reduction of mean observed duration from onset to hospital admission (January 27) and the implementation of travel restriction within the city (early February). Because of the lack of information, we assumed that early mean duration from onset to hospital admission (before January 27) is close to that in Wuhan. Finally, we also performed sensitivity analysis based on the mean interval incubation (S1 Fig in S1 File).

## Results

### Epidemiological feature of COVID-19 in Wenzhou

**Fig 2:**
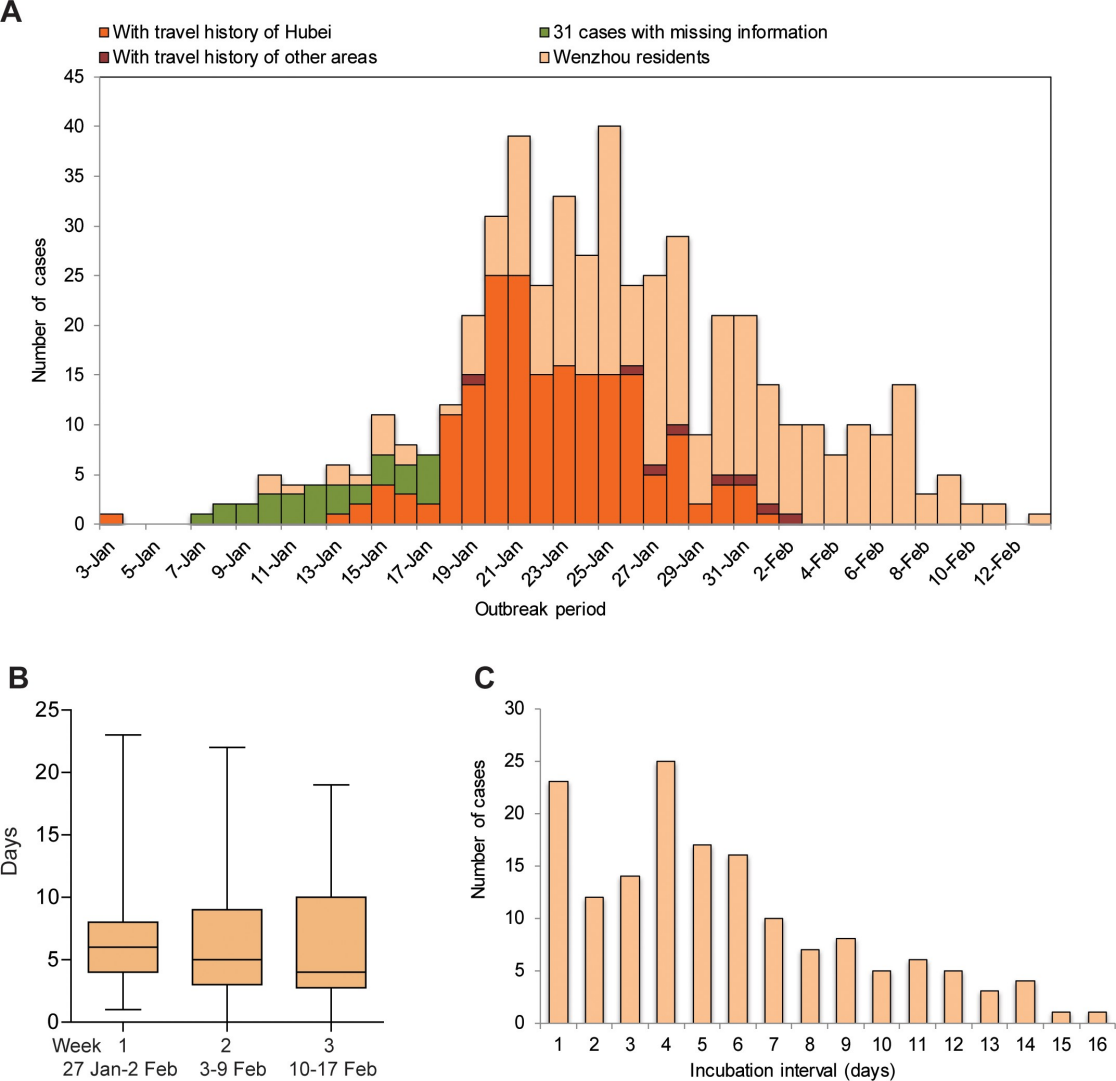
Epidemical features of COVID-19 in Wenzhou. (A) Distribution of disease onset of all the identified cases. Because specific information of the early 31 imported cases is not available; we assumed that date of onset of all imported cases follows a Gaussian distribution, and arbitrarily ranged them in the period from January 7 to 17. (B) Information on duration from onset to hospital admission of 467 cases from January 27 to February 17. (C) Distribution of interval of incubation of 157 imported cases.

We systematically collected the epidemiologic information of in total 504 cases which were identified in Wenzhou by March 5, 2020 (Table 1, Fig 1B). We first analyzed the data based on the distribution of onset date. Because this information was missing for the early 31 cases identified from January 21 to 26, we assumed that the onset of these cases follows a Gaussian distribution and re-ranged the distribution of the 31 cases between January 7 and 17 (Fig 2A). The duration from onset to hospital admission was continuously decreased over the periods from January 3 to 20 (reported 20 days; Table 1), January 21 to 27 (assumed 12 days; Table 1), and then became stable as observed 6 days in average (Fig 2B), until there was no new case identified. Based on the available information of imported cases, the average incubation interval was estimated as 5.5 (95% CI, 4.8–6.2) days (Fig 2C).

### Modeling control measures in Wenzhou

How rapid an epidemic can spread largely depends on the reproductive number. The basic reproductive number, *R*_*0*_, is defined as the number of secondary infections generated by one primary case in a totally susceptible population. The effective reproductive number, *R*, measures the number of secondary cases generated by an index case and the declines because of the reduction of susceptibility in the population and the use of effective control measures. *R* must be below 1 to stop an outbreak. The epidemic of Wenzhou was mainly caused by imported cases with travel history of Wuhan or other areas of China, which accounts for 43.7% (Fig 2A). The value of *R*_*0*_ is estimated to be 2.3 (95% CI, 2.2–2.5).

**Fig 3:**
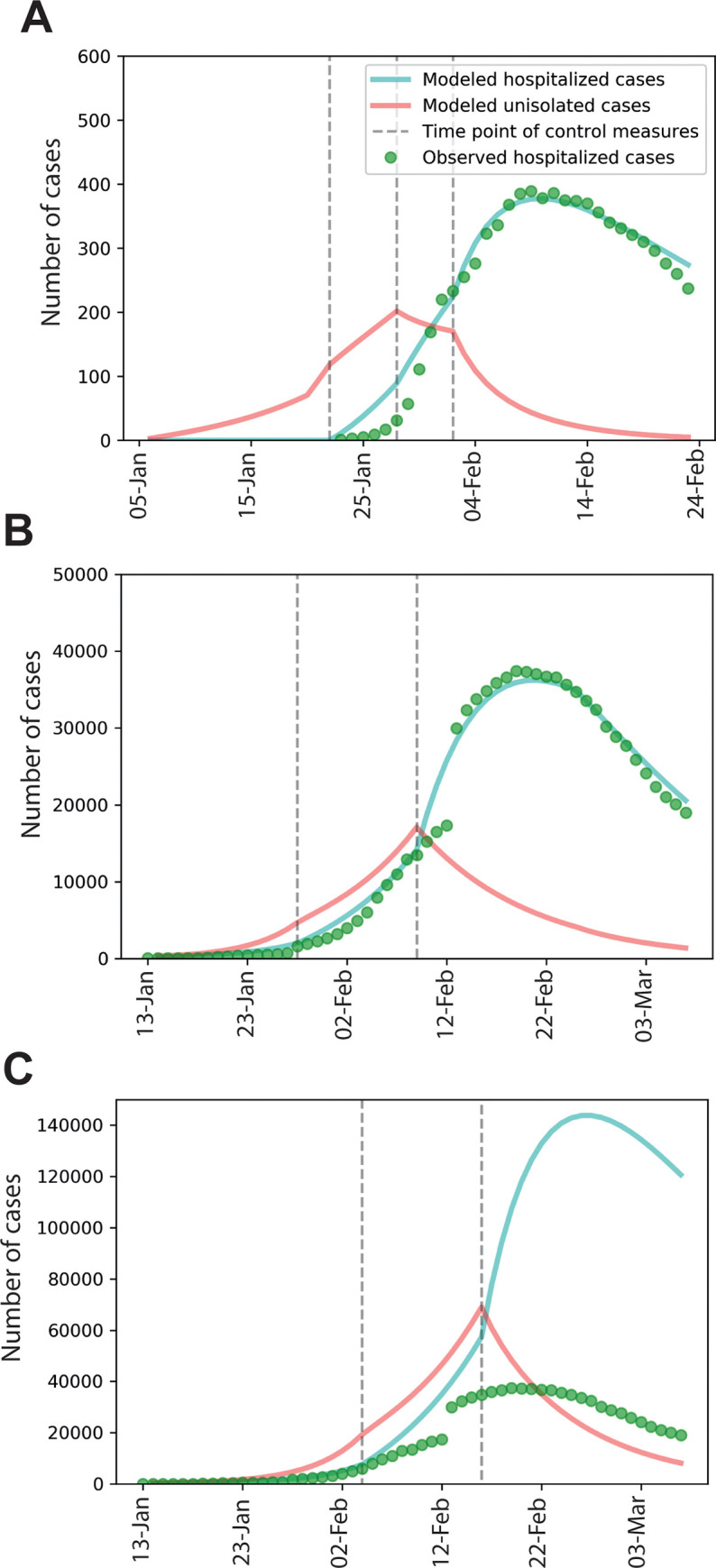
Simulation of COVID-19 epidemics in Wenzhou and Wuhan. (A) Modeled hospitalized and unidentified cases in Wenzhou from January 3 to February 21. The first dash line is on January 21 when the first case was identified. The second dash line is on January 27 when mean duration from disease onset to hospital admission reduced from 12 days to observed 6.1 days. The last dash line is on February 2 when a within-city quarantine started. (B) Modeled hospitalized and unidentified cases in Wuhan from January 15 to March 5. The first dash line is on January 26 when a within-city quarantine started. The second dash line is on February 10 when temporary emergency units were operational. (C) Modeled hospitalized and unidentified cases in Wuhan with a seven-day delay of the two control measures of (B).

**Fig 4:**
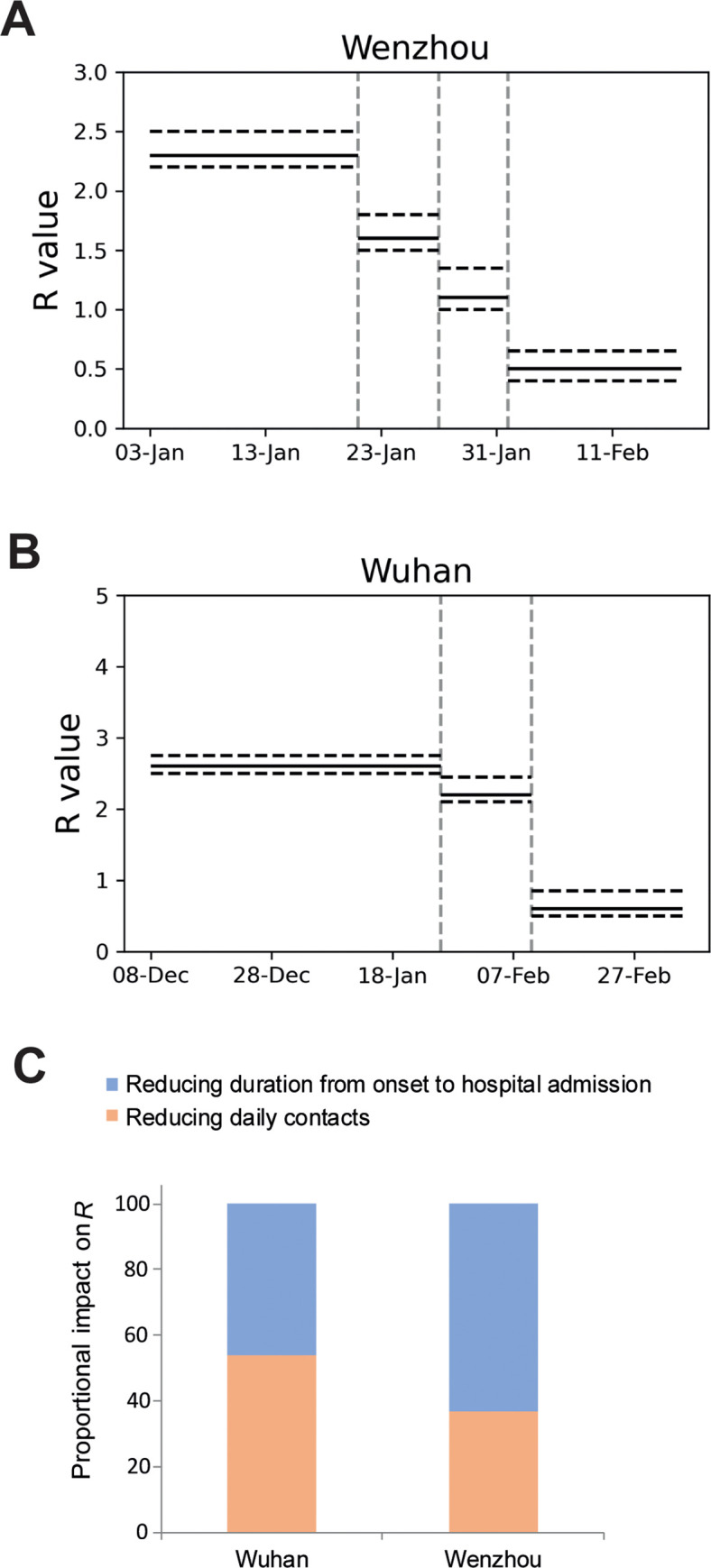
Estimating effective reproductive number *R*. Estimated reproduction number for the period of the epidemic in Wenzhou (A) and Wuhan (B). Confidence intervals are also shown. (C) Proportion of impact of reducing contact rate and duration from onset to hospital admission to *R* values.

Our mathematical model has well-reproduced the COVID-19 epidemic with several key epidemic patterns identified (Fig 3). The unidentified cases were initially composed of only imported cases, but as the transmission proceeding more secondary cases from the local were gradually included. Importantly, the model shows that the epidemic has been fully contained and the number of unidentified cases is close to zero by February 17. This perfectly matches the real-world situation that no new cases were further identified. Since the first case identified on January 21, mean duration from disease onset to hospital admission were continuously decreased. This is important for preventing further spread by infected cases. Correspondingly, the modeled *R* values were reduced to 1.6 and 1.1 (Fig 4A). Finally, it reached 0.5 after implementation of within-city quarantine, which has dramatically reduced average daily contacts from initial 10 to 6.

### Modeling epidemiology and control measures in Wuhan

In the model of Wuhan epidemic, we have systematically collected the epidemiological data (S1 Table in S1 File; Fig 1B). We found that both within-population quarantine and the use of emergency unit are effective (Fig 3B). After the first time point (January 26), modeled within-population contact rate was decreased from initial 10 to 6, and then 3.5 after the second time point (February 10). A seven-day delay of implementing these two measures would exacerbate the epidemic, and indirectly increase the burden of quarantine (Fig 3C). Our model estimated 983 unidentified or unisolated cases by March 5, 2020. According to the current effectiveness, it requires additional 40 days to contain all these unidentified cases.

According to our modeling, prior to applying effective control measures, the estimated number of unidentified or unisolated cases was always higher than that of hospitalized cases. Within-population quarantine started on January 26 was proven to be effective, reducing *R* from the estimated initial value of 2.6 to 2.2 (Fig 4B). After February 10, many of these hidden cases were isolated at the new emergency units temporarily built for this epidemic. Correspondingly, the modeled mean duration from onset to hospital was reduced from 10 to 3.2 days after February 10. Modeled *R* value within this period is about 0.6 (95% CI, 0.5–0.8) (Fig 4B), indicating the feasibility of containing the epidemic.

### Comparative analyses between Wuhan and Wenzhou

The initiation, development and eventual outcomes of the epidemics in these two cities are evidently distinct. Before adopting within-population quarantine and travel restriction, the epidemic has already developed for more than one month (about 49 days) in Wuhan (Fig 2B). In addition to 630 hospitalized cases, the number of the unidentified cases was estimated to be around 5,000 by January 26 (Fig 3B).

By striking contrast, the response period in Wenzhou is much shorter. Before adopting quarantine, the estimated number of unidentified case is 180 by February 2. This relatively low number allowed subsequent control by rapid allocation of medical resources per capita from the local authorities. Effectiveness of quarantine and reduction of the period for disease onset to hospital admission was compared in the construct of *R* (Fig 4C). Quarantine contributes to 37% and reduction of duration from onset to hospital admission accounts for 63% to the effectiveness in Wenzhou. In Wuhan, Quarantine contributes to 54% and the other accounts for 46%, respectively (Fig 4C).

## Discussion

The transmission dynamics of a virus is primarily determined by the effective reproductive number. These *R* values often constantly evolve attributing to viral adaption, susceptibility of the targeting population, environmental changes, implementation of control measures. Although the basic reproductive number *R*_*0*_ of SARS-CoV-2 (2.6) appears to be even lower than that of SARS-CoV (2.9) [8], the speed and scale of COVID-19 spreading greatly surpass SARS and MERS [9]. The drastic escalation of the epidemic in Wuhan mainly attributed to the non-responsiveness of the local authorities at the early stage. Incredibly, a massive annual potluck banquet for 40,000 families was continued to be held in Wuhan on January 18, which very likely exacerbated the outbreak. Mass gatherings could impose high risks of super-spreading event (SSE) [10]. We suspected that SSE likely occurred in Wuhan at the early stage. However, we do not have actual data to confirm the occurrence of SSE, and therefore it is not specifically emphasized in our model.

The turning point for coping with the epidemic in Wuhan was the implementation of vigorous public health measures directly ordered by the central government. From January 23, the central government began to implement heavy control measures, including city lockdown, travel ban, and within-population quarantine. The implantation of travel ban of Wuhan was already too late to have major impact on the spread in Mainland China, although it was estimated to have substantial impact at the international scale [11]. In our analysis, we emphasized two important factors that determine the outcomes in containing the epidemic including effectiveness of quarantine, and the duration from disease onset to hospital admission. A major challenge for Wuhan was that the numbers of confirmed and suspected COVID-19 cases were already too high at the time of implementing measures. This has plunged the local healthcare system into crisis, and all the resources have to exclusively dedicate to contain this outbreak. Since late January and early February, the central government has mobilized strong medical forces and emergency medical supplies, and new emergency units were built in very short-term. These efforts have leveraged the levels of quarantine, and shortened the duration from disease onset to hospital admission that prevented spreading by infected cases.

One important reason for the wide and quick spread of COVID-19 to other parts of China is that the Wuhan outbreak coincided with the Chinese lunar new year holiday. It is a massive population migration with estimated 3 billion individual trips to take place [2]. Wuhan is a large hub connecting different parts of China through railways and an international airport, and five million people have left Wuhan before the travel ban [12]. A heavily affected city in other part of China is Wenzhou, because there are an estimated 170,000 Wenzhou businesspeople working in Wuhan [3]. Because of the returnees from Wuhan, Wenzhou had the highest figure of imported COVID-19 cases for any city outside Hubei province [3]. However, it only took 46 days to fully contain the epidemic, otherwise bearing high risk of growing into a new epicenter. Based on our analysis, the key to achieve this outcome is attributed to the quick response from the local authorities and the general public. The government has rapidly implemented restrictive measures including city closure, travel ban, effective isolation and quarantine, and rapid case identification and hospital admission. The public is highly aware of the contagiousness of SARS-CoV-2, and spontaneously adopt social distancing measures such as cancellation of public gatherings and holiday visits, and minimizing outdoor activities. This resulted in continuous decrease of *R* values to 1.6, 1.1 and 0.5, and no new case was further reported in Wenzhou.

Given the nature of retrospectively analyzing the outcomes of the COVID-19 epidemics, our findings are not of completely unexpected. However, we provided quantitative analysis of the impact of reducing duration from disease onset to hospital admission and within-population quarantine. Importantly, we have comparatively taken two real-world examples with unique characteristics as a COVID-19 epicenter and a setting with mainly imported cases. These findings would add new insight in understanding control measures at early stages of the COVID-19 epidemics, and shall serve as an importance reference for quick response to future new outbreaks/epidemics with full consideration of the real-world situations.

Because of the disparities in culture, socioeconomic status and types of government, the awareness, preparedness and responsiveness towards emerging threats vary dramatically among different countries [9, 13–17]. Our results highlight the essential of early, quick and adequate response with implementation of the right control measures. Delayed response will soon plunge the healthcare system into crisis, as seen in Wuhan and other countries like Italy, Spain, UK and USA, even with well-developed healthcare systems. Availability of healthcare is critical for controlling the epidemic but also for minimizing severe patient outcomes [18]. As seen in Wenzhou, rapid response has avoided to emerge as a new epicenter, and soon contained the epidemic. On the other hand, understanding and cooperation from the general public are also extremely important for the effectiveness of these control measures [19].

In summary, we comparatively analyzed the epidemiology, estimated the transmission dynamics, and assessed the effectiveness of control measures between Wuhan and Wenzhou by monitoring the dynamics of the effective reproductive number *R*. Our findings suggest that combination of reducing the interval from disease onset to hospital admission and stringent within-population quarantine are effective for both epicenters and regions mainly caused by imported infections. These two cities in China represent real-world examples that can serve as important lessons for improving preparedness and early response to future emerging epidemic/pandemic.

## Supporting information

S1 File (DOCX)

## Abbreviations

COVID-19: coronavirus disease 2019
CI: confidence interval
SSE: super-spreading events
SARS-CoV-2: severe acute respiratory syndrome coronavirus 2
SEIR: susceptible-exposed-infectious-recovered
R: effective reproductive number
R_0_: basic reproductive number
