# Supplementary material for: Distinct effectiveness in containing COVID-19 epidemic: Comparative analysis of two cities in China by mathematical modeling
Source: PLOS Glob Public Health. 2021 Nov 12;1(11):e0000043. doi: 10.1371/journal.pgph.0000043 (PMC10021246; doi:10.1371/journal.pgph.0000043)
Supplement: S1 File — (DOCX) [file pgph.0000043.s001.docx]

**Supplementary Material**

1. Transmission model for the effect of quarantine

The model depicted in Figure 2 consists of the following ordinary differential equations (Lipsitch, Cohen et al. 2003).

${dS}/{dt}=-cbI_{u}X/N+rS_{q}$ (1)

$d{S_{q}}/{dt}=qc\left( 1-b \right)I_{u}S/N-rS_{q}$ (2)

$dE/{dt}=-pE+cb(1-q)I_{u}S/N$ (3)

$d{Eq}/{dt}=qcbI_{u}S/N-pE_{q}$ (4)

$d{I_{u}}/{dt}=pE-(v+m+i)I_{u}$ (5)

$dH/{dt}=iso\left( I_{u}+I_{q} \right)-(v+m)H$ (6)

$d{I_{q}}/{dt}=pE_{q}-\left( v+m+i \right)I_{q}$ (7)

$dR/{dt}=v(I_{u}+H+I_{q})$ (8)

$dD/{dt}=m(I_{u}+H+I_{q})$ (9)

The model (including equations (1) to (9)) is a modification of the classical SEIR model, involving susceptible, infected but not yet infectious, infectious, and recovered/immune individuals in compartments *S, E, I* and *R* respectively. At time *t*=0, there are *N* people in the population, of whom certain number of individuals are exposed but not yet infectious (*E*), and all the rest are susceptible (*S*). In our model, the *I* compartment is composed of cases who are not isolated (*I_u_*) and those who have been isolated (*H*). *c* is the number of daily contacts per person, and b is the probability of transmission per contact between a susceptible and an infectious person. We separated the parameters *c* and *b*, rather than using transmission parameter, *β*. We thus assume that each infectious person makes *c* contacts per day, of whom a proportion *b* are infected if the infectious person is undetected. We attained b based on the function $R=\beta\times mean duration of infectiousness$. 1/*r* is the mean duration of quarantine for susceptible persons who are suspected to be infected but actually not (*S_q_*), they would flow back to susceptible (*S*). 1/*p* is the mean time for progression from latent to infectious. *v* is the per capita recovery rate, *m* is the per capita death rate, and *i* is the mean daily rate at which infectious cases are detected and isolated. In our model, we calculated *i* as the mean rate of cases from onset to hospital admission.

We made a simplified assumption that isolated cases (*I_d_*) do not infect anyone, and the rate of isolation *i* should be thought of as a rate of “effective” isolation. Under these assumptions, the mean duration of infectiousness is $1/(v+m+i).$ We estimated parameters by using least-square fitting to look for the model trajectory that best matches the epidemic time series. Specifically, we fit the hospitalized number of cases given by equation *H(t)* to the hospitalize number of case notifications (Figure 4A and B).

For epidemic in Wenzhou, we modified equation (5) as:

$d{I_{u}}/{dt}=pE-\left( v+m+i \right)I_{u}+import$ (5’),

where *import* is mean number of onset of imported cases with travel history of Hubei province or other areas (Figure 3A). At time *t*=0, we assumed that all the individuals in the model are susceptible, and imported infectious cases enter the model as rate of *import*.

2. Estimating R_0_

We used a time-dependent method to compute *R_0_* (Obadia, Thomas et al. 2012; Wallinga, Jacco et al. 2004). Briefly, the probability *p_ij_* that case *i* was infected by case *j* is presented as

$p_{ij}=\frac{N_{ij}w(t_{i}-t_{j})}{\sum_{i\neq k} N_{i}w(t_{i}-t_{k})}$,

Where *t_i_* is the onset time of case *i*, and *k* denotes the index of other case that are primary case of case *i*. *w(t)* is the generation interval, or the serial interval, which is the time from symptom onset of a primary case to that of its secondary case. As generation interval was not directly observed in this study, we assumed that the timing of transmission events is not skewed during the early term of the infectious period, and approximately calculated the interval as sum of the average incubation period and half of the average infectious period (Fine, Paul et al 2003). *N_ij_* is the number of case *i* infected by case *j*, and *N_i_* is the overall number of case *i.* The reproduction number for case *j* is the sum over all case *i*, weighted by the probability that case *i* is infected by case *j*:

$R_{j}=\sum_{i} p_{ij}$,

and reproduction number over all cases with the same date of onset is

$R_{t}=\frac{1}{N_{t}}\sum_{\left\{ t_{j}=t \right\}} R_{j}$.

Combined with equation derivation above, we calculated the basic reproduction number *R_0_* by determining the initial exponential disease growth phase with the goodness-of-fit test statistic (Lipsitch, Marc et al. 2003), and obtained the confidence interval by model simulation.

3. Estimating time-dependent R

We used a simple method to calculate the effective reproduction number *R_t_* with real-time data (Contreras, Sebastián et al. 2020). Briefly, in a standard *SEIR* model, four compartments were established, namely, susceptible individuals *S*, exposed individuals *E*, infected individuals *I*, and removed individuals *R*. The dynamics of the *SEIR* model are thus represented by:

$$\begin{aligned} \acute{S}= -\frac{\beta\left( t \right)SI}{N}\#\left( 1 \right) \end{aligned}$$

$$\begin{aligned} \acute{E}=\frac{\beta\left( t \right)SI}{N}-\sigma\left( t \right)E \#\left( 2 \right) \end{aligned}$$

$$\begin{aligned} \acute{I}= \sigma\left( t \right)E- \gamma\left( t \right)I-\mu\left( t \right)I\#\left( 3 \right) \end{aligned}$$

$\begin{aligned} \acute{R}= \gamma\left( t \right)I\#\left( 4 \right) \end{aligned}$.

We combined equations (1), (2) and (3), and attained:

$\begin{aligned} \frac{dI}{dS}+\frac{dE}{dS}=-1+\frac{\left( \gamma\left( t \right)+\mu\left( t \right) \right)}{\beta\left( t \right)}\frac{N}{S}\#\left( 5 \right) \end{aligned}$.

We thus calculate *R_t_* as the ratio between the time-dependent infection, and combination of recovery rates and case fatality rate, $\beta(t)$, $\gamma\left( t \right)+\mu\left( t \right)$ (Chen, Yi-Cheng et al. 2020), multiplied by the proportion of susceptible individuals in the whole population ($\frac{S}{N}$):

$$\begin{aligned} R_{t}\left( t \right)= \frac{\beta\left( t \right)}{\left( \gamma\left( t \right)+\mu\left( t \right) \right)}\frac{N}{S}\#\left( 6 \right) \end{aligned},$$

and equation (5) can be re-written as:

$\begin{aligned} \frac{dI}{dS}+\frac{dE}{dS}=-1+\frac{1}{R_{t}\left( t \right)}\#\left( 7 \right) \end{aligned}$.

Equation (7) can be discretized in an interval $\left[ t_{i-1},t_{i} \right]$ where we can assume that $R_{t}\left( t \right)=R_{t}\left( t_{i} \right)$ is constant:

$\begin{aligned} R_{t}\left( t_{i} \right)=\frac{1}{\frac{\Delta_{i}I}{\Delta_{i}S}+\frac{\Delta_{i}E}{\Delta_{i}S}+1}\#\left( 8 \right) \end{aligned}$.

Extending the classic SEIR model to consider also exposed individuals and deaths, a disease states balance dictates the discrete differences to follow $\Delta_{i}S+\Delta_{i}E+\Delta_{i}I+\Delta_{i}R+\Delta_{i}D=0$. Then, Equation (8) takes the final form applying the chain rule:

$\begin{aligned} R_{t}\left( t_{i} \right)=\frac{1}{1-\frac{\Delta_{i}E+\Delta_{i}I}{\Delta_{i}E+\Delta_{i}I+\Delta_{i}R+\Delta_{i}D}}\Longleftrightarrow R_{t}\left( t_{i} \right)=\frac{\Delta_{i}E+\Delta_{i}I}{\Delta_{i}R+\Delta_{i}D}+1\#\left( 9 \right) \end{aligned}$.

Based on equation (9), we estimated *R_t_* with real-time data by model simulations.

Table S1. Parameters used in the quarantine simulation of Wuhan epidemic

| Parameter | Symbol | Time | Baseline value | Estimated value | Reference |
| --- | --- | --- | --- | --- | --- |
| Contact rate | *c* | 15-26 Jan | 10 | - | Assumption |
|  |  | 27 Jan-9 Feb | - | 6 | Estimated |
|  |  | 10 Feb-6 Mar | - | 3 | Estimated |
| Mean duration from onset to hospital admission | *i* | 15-26 Jan | 10 days  (9-12) | - | (Li, Guan et al. 2020) |
|  |  | 27 Jan-9 Feb | - | 10 days (9-12) | Estimated |
|  |  | 10 Feb-5 Mar | - | 3.2 days (3-3.8) | Estimated |
| Mean Recovery rate | *v* | - | 0.0237  (0.02-0.041) | - | the Health Commission of Wuhan |
| Mean interval of incubation | *p* | - | 4 days  (3.8-7) | - | (Special Expert Group for Control of the Epidemic of Novel Coronavirus Pneumonia of the Chinese Preventive Medicine 2020) |
| Mean case fatality rate | *m* | - | 0.0324  (0.031-0.044) | - | (Special Expert Group for Control of the Epidemic of Novel Coronavirus Pneumonia of the Chinese Preventive Medicine 2020) |

4. Sensitivity of mean interval incubation

Based on most recent studies on COVID-19, mean interval of incubation is around 3 to 4 days, but its range is large, and there is clinical case that the largest interval could reach 24 days. Therefore we analyzed the sensitivity of interval of incubation to the simulation (Figure S1). It shows that even mean interval of incubation is reduced to 2 days, the epidemic would be stopped although a higher medical burden will be imposed.


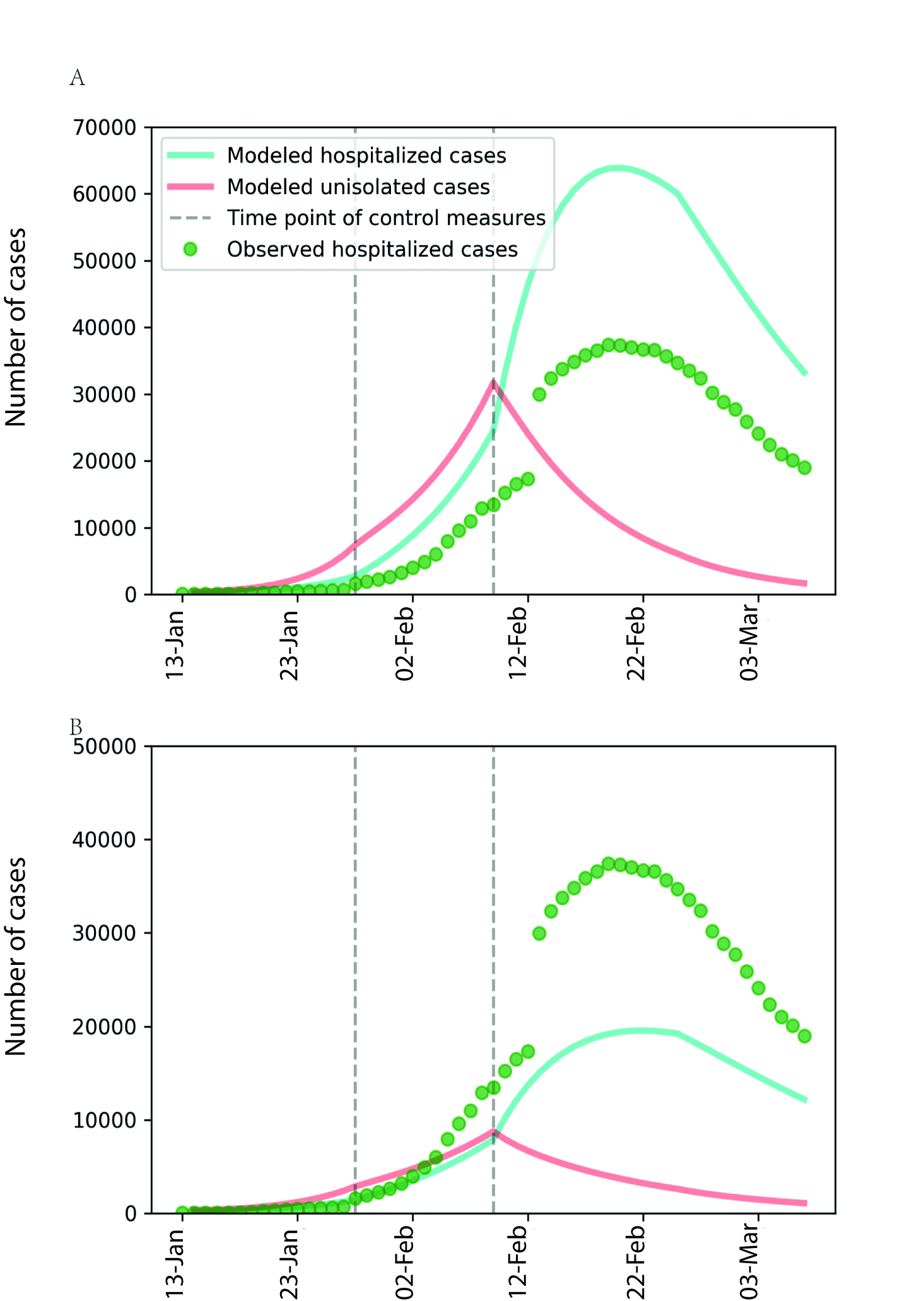


Figure S1. Sensitivity analysis of mean interval of incubation to the simulation of Wuhan epidemic. (A) Mean interval of incubation is assumed to 2 days. (B) Mean interval of incubation is assumed to be 6 days.

References

Obadia, T., et al. (2012). “The R0 package: a toolbox to estimate reproduction numbers for epidemic outbreaks.” BMC Med Inform Decis Mak**12**:147.

Wallinga, J., et al. (2004). “Different epidemic curves for severe acute respiratory syndrome reveal similar impacts of control measures.” Am J Epidemiol **160** (6):509-516.

Fine, PE. (2003). “The interval between successive cases of an infectious disease.” Am J Epidemiol**158**(11):1039-47.

Lipsitch, M., et al. (2003). "Transmission dynamics and control of severe acute respiratory syndrome." Science **300**(5627): 1966-1970.

Contreras, S., et al. (2020). “Real-Time Estimation of Rt for Supporting Public-Health Policies Against COVID-19.” Front Public Health **8**:556689

Chen, YC., et al. (2020). “A Time-Dependent SIR Model for COVID-19 With Undetectable Infected Persons." Transactions on Network Science and Engineering**7**(4): 3279-3294

Li, Q., et al. (2020). "Early Transmission Dynamics in Wuhan, China, of Novel Coronavirus-Infected Pneumonia." N Engl J Med.

Lipsitch, M., et al. (2003). "Transmission dynamics and control of severe acute respiratory syndrome." Science **300**(5627): 1966-1970.

Special Expert Group for Control of the Epidemic of Novel Coronavirus Pneumonia of the Chinese Preventive Medicine, A. (2020). "[An update on the epidemiological characteristics of novel coronavirus pneumoniaCOVID-19]." Zhonghua Liu Xing Bing Xue Za Zhi **41**(2): 139-144.
